# Toxicity Assessment of a Single Dose of Poly(ethylene glycol) Diglycidyl Ether (PEGDE) Administered Subcutaneously in Mice

**DOI:** 10.3390/toxics9120354

**Published:** 2021-12-15

**Authors:** Do-Hyun Kim, Jong-Hyeon Han, Hyuk-Cheol Kwon, Su-Jin Lim, Seo-Gu Han, Hyun-Su Jung, Keyong-Ho Lee, Ju-Hee Kang, Sung-Gu Han

**Affiliations:** 1 Toxicology Laboratory, Sanghuh College of Life Science, Konkuk University, 120 Neungdong-ro, Seoul 05029, Korea; secret311@konkuk.ac.kr (D.-H.K.); hyeon4970@konkuk.ac.kr (J.-H.H.); rnjs1024@konkuk.ac.kr (H.-C.K.); tnwlsdl1110@konkuk.ac.kr (S.-J.L.); tjrn8854@konkuk.ac.kr (S.-G.H.); jehceh@konkuk.ac.kr (H.-S.J.); 2 R&D Department, Across Co., Ltd., Chuncheon 24398, Korea; keyho625@across.kr (K.-H.L.); jhkang@across.kr (J.-H.K.)

**Keywords:** Poly(ethylene glycol) diglycidyl ether, subcutaneous toxicity, epidermal ulcer, dermal wound

## Abstract

Poly(ethylene glycol) diglycidyl ether (PEGDE) is widely used to cross-link polymers, particularly in the pharmaceutical and biomaterial sectors. However, the subcutaneous toxicity of PEGDE has not yet been assessed. PEGDE samples (500–40,000 μg/mouse) were subcutaneously injected into the paraspinal dorsum of BALB/c male mice. Cage-side observations were carried out with measurement of organ weight, body weight variation, and feed intake, as well as histopathological characterization on day 28 post-exposure. Mice that received 40,000 μg of PEGDE showed severe toxic response and had to be euthanized. Subcutaneous injection of PEGDE did not alter feed intake and organ weight; however, the body weight variation of mice injected with 20,000 μg of PEGDE was significantly lower than that of the other groups. Exposure to 10,000 and 20,000 μg of PEGDE induced epidermal ulcer formation and hair loss. The histology of skin tissue in mice administered with 20,000 μg of PEGDE showed re-epithelialized or unhealed wounds. However, the liver, spleen, and kidneys were histologically normal. Collectively, PEGDE, particularly above 10,000 μg/mouse, caused subcutaneous toxicity with ulceration, but no toxicity in the other organs. These results may indicate the optimal concentration of subcutaneously injected PEGDE.

## 1. Introduction

Poly(ethylene glycol) diglycidyl ether (PEGDE) is a water-soluble chemical used to cross-link polymers containing hydroxyl, amine, and carboxyl groups [1]. PEGDE is also used for surface modification, to combine a hydroxy group with a protein, and in membrane technology [2,3,4]. Because of its low toxicity, PEGDE has been applied in pharmaceutical and biomaterial sectors [5,6,7]. For example, PEGDE was used to make injectable hyaluronic acid (HA)-based hydrogels, which were injected subcutaneously to improve soft-tissue deficits in the skin [6,8]. In addition, PEGDE was used as a cross-linker for HA-itaconic acid film which is a bioadhesive matrix used for topical drug delivery in ophthalmology [9]. Further, PEGDE crosslinked HA fillers were utilized for facial rejuvenation and aesthetic enhancement following facial reconstructive surgery [10]. In such cases, PEGDE is used as a cross-linking agent to allow the formation of C-O-C bonds, thus increasing the stability of a hydrogel. Another cross-linking agent available as a component of biomaterials for hydrogels is 1,4-butanediol diglycidyl ether (BDDE). In HA hydrogels, cross-linking agents used for the formation of stable polymers can remain as unreacted or residual cross-linkers. Because the residual cross-linker is a by-product generated during the manufacturing process of polymers, it is necessary to remove the residues from the final product [11]. The remaining cross-linker residues can exhibit toxicity at high concentrations [12,13]. These cross-linking agents have been reported to induce cytotoxicity. For example, BDDE induced contact allergy in a patch test and acted as an allergen in brush factory workers [14,15]. In contrast, PEGDE-treated human keratinocyte cells and human dermal fibroblast cells exhibited higher levels of cell viability, lower extent of cell membrane disruption, lower levels of cellular oxidative stress, and more muted inflammatory response than BDDE-treated cells [16]. This previous study provided in vitro evidence that PEGDE induces lower levels of dermal toxicity than BDDE. However, toxicology data obtained from experimental animals that were subcutaneously exposed to PEGDE are unavailable. Therefore, this study aimed to test the subcutaneous toxicity of PEGDE in mice. In the current study, the highest dose level was selected as 40,000 μg, and lower doses (0, 500, 1000, 5000, 10,000, and 20,000 μg) were selected to investigate dose-response relationships. These doses were selected based on our preliminary study where mice injected with 10,000 μg of PEGDE showed no clinical symptoms for 28 days, whereas mice injected with 100,000 μg of PEGDE died 2–3 h after injection. In the current study, PEGDE was injected subcutaneously into male BALB/c mice, and mouse tissue samples, including the skin, liver, kidney, and spleen, were examined for local and systemic toxic responses.

## 2. Materials and Methods

### 2.1. Test Substances

PEGDE (average M_n_ = 500, CAS: 26403-72-5) was purchased from Sigma-Aldrich (St. Louis, MO, USA). PEGDE was diluted in phosphate-buffered saline (PBS) at concentrations of 0–40,000 μg/200 μL PBS and filtered through a 0.2-μm syringe filter (Millipore, MA, USA). The PEGDE sample was prepared prior to injection in mice.

### 2.2. Experimental Animals

All experiments with mice were conducted following the regulations of the Institutional Animal Care and Use Committee (IACUC) of the Ethics Committee of Konkuk University (Seoul, Korea; IACUC No. KU21089). The animals were handled humanely according to the animal welfare guidelines issued by the Korean National Institute of Health and the Korean Academy of Medical Sciences for the care and use of laboratory animals. Six-week-old BALB/c male mice (20–24 g body weight) were obtained from Orient Bio Inc. (Seoul, Korea). The mice were housed in polycarbonate cages with beta chip^®^ (Northeastern Products Corp., Warrensburg, NY, USA) bedding at room temperature (22 ± 2 °C) and constant humidity (40–70%) with 12 h light-dark cycles. Mice were provided a standard 5L79 diet (Orient Bio Inc., Seoul, Korea) and water ad libitum throughout the experiments. All mice were acclimatized for a week before subcutaneous injections of the test substances.

### 2.3. Experimental Design

Six-week-old BALB/c male mice were randomly grouped into PBS (control), PEGDE 500 μg/mouse, PEGDE 1000 μg/mouse, PEGDE 5000 μg/mouse, PEGDE 10,000 μg/mouse, PEGDE 20,000 μg/mouse, and PEGDE 40,000 μg/mouse groups (*n* = 5 mice/group). The details of the mouse treatments are presented in Table 1. Two hundred microliters of PBS (control) or PEGDE solution (500–40,000 μg/200 μL PBS/mouse) were injected subcutaneously into the paraspinal dorsum of the mice using a medical syringe. On day 28 after the injection, mice were anesthetized by intraperitoneal injection of 2.5% tribromoethanol (Sigma-Aldrich; T48404), and the skin, liver, spleen, and kidneys were collected and weighed.

#### 2.3.1. General Clinical Observations

After sample injection, the survival rate (%) of mice was measured for 24 h. Body weight variation (%) and feed intake (g/day/mice) were recorded before injection (day 0) and after injection (days 7, 14, 21, and 28). Photographs were taken after the development of the wound on the paraspinal dorsum of the mice (days 10, 13, 17, 20, and 27).

#### 2.3.2. Absolute and Relative Organ Weights

Absolute weights (g) of organs (liver, spleen, and kidney) were measured for each mouse. In addition, the relative organ weight (% of body weight) was calculated using the following Formula (1):

Relative organ weight (% of body weight) = organ weight (g)/mouse body weight (g) × 100(1)

#### 2.3.3. Cage-Side Observations

Mice were continuously monitored for the first 30 min after sample injection, followed by every 30 min until 24 h, and then every day for 28 days. Mortality, morbidity, appearance, behavior pattern, gait, fur condition, and breathing abnormalities were observed throughout the experimental period. Clinical signs were graded for severity, and grades were coded as none (grade 0), slight (grade 1), moderate (grade 2), severe (grade 3), or very severe (grade 4). For mortality, only absence (grade 0) or presence (grade 1) of death was scored.

#### 2.3.4. Histological Analysis

After euthanasia, all tissues (skin, liver, spleen, and kidneys) were fixed in a 4% buffered formaldehyde solution for histological analysis. The tissues were dehydrated using graded ethanol and embedded in paraffin. Paraffin-embedded tissues were cut into 4 μm sections using a blade (Feather Microtome Blade A35), deparaffinized with xylene, rehydrated with graded ethanol, and stained with hematoxylin-eosin (H&E). Tissue images were digitally captured using an Olympus DP71 camera and DP software (Olympus Optical Co., Ltd., Tokyo, Japan), as described previously [17].

### 2.4. Statistical Analysis

Data on body weight variation, feed intake, and organ weight are presented as the mean ± standard error (SE). Data were analyzed for statistical significance using SPSS PASW statistical software, version 18.0 for Windows (SPSS, Chicago, IL, USA). All *p*-values were estimated by one-way ANOVA followed by Tukey’s post hoc test and independent two-sample *t*-test. Statistical significance was set at *p* < 0.05.

## 3. Results

### 3.1. Clinical Observation and Skin Injury

**Figure 1:**
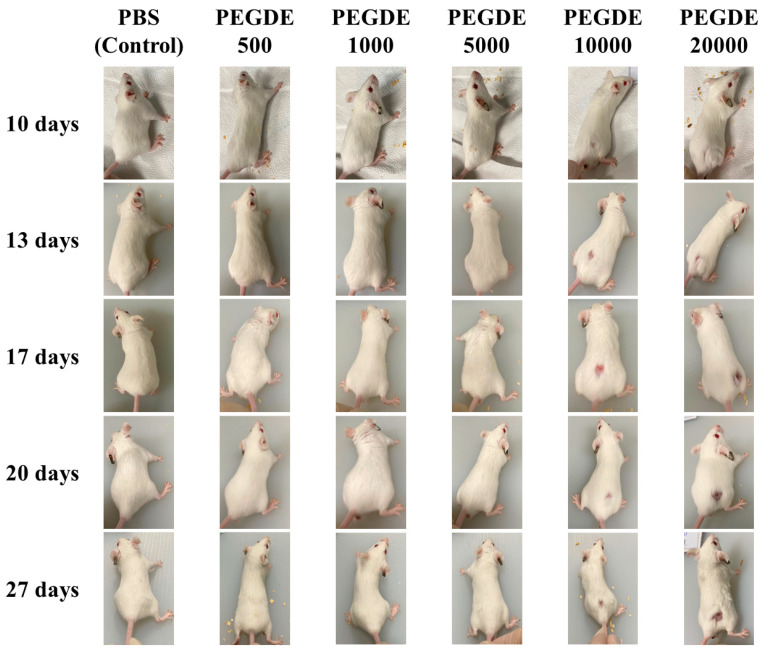
Photograph of representative mice injected with Poly(ethylene glycol) diglycidyl ether (PEGDE). Photographs of mice were taken on days 10, 13, 17, 20, 27 after PEGDE injection at a standard distance. Seven-week-old BALB/c male mice were grouped into PBS (control), PEGDE 500 μg/mouse, PEGDE 1000 μg/mouse, PEGDE 5000 μg/mouse, PEGDE 10,000 μg/mouse, and PEGDE 20,000 μg/mouse groups (*n* = 5 mice/group).

To evaluate clinical abnormalities, clinical signs and symptoms of the mice were monitored carefully throughout the experimental period. Mice injected with 0–20,000 μg of PEGDE (PBS, P1-P5) showed no abnormalities in morbidity, appearance, behavior pattern, gait, fur condition, or breathing pattern until 24 h after the injection (Table 2). However, mice injected with 40,000 μg of PEGDE exhibited impairment of hind leg movement, breathing abnormalities, poor movement, and gait abnormalities from 3 h after the injection (Table 2). Therefore, the exposed group was euthanized. No other mice were euthanized based on clinical observations during the 28-day observation period. The development of hair loss and skin ulcer was observed at the injection site 10 days after injection at doses of 10,000 and 20,000 μg of PEGDE (Figure 1). In 10,000 μg PEGDE-injected mice, the ulcer and scar became larger until 17 days after the injection and then recovered on days 20 and 27, while hair loss at the injection site was not fully recovered. Mice injected with 20,000 μg of PEGDE exhibited severe scarring and hair loss on day 17, which slowly recovered but remained until the termination of the experiment (day 28). In addition, in mice injected with 20,000 μg PEGDE, the fur was duller than that of the 10,000 μg PEGDE-injected group. Overall, the results demonstrated that subcutaneous injection of 10,000 μg or more of PEGDE could cause skin injury, hair loss, and the appearance of dull fur at the injection site.

### 3.2. Feed Intake, Body Weight, and Organ Weight

**Figure 2:**
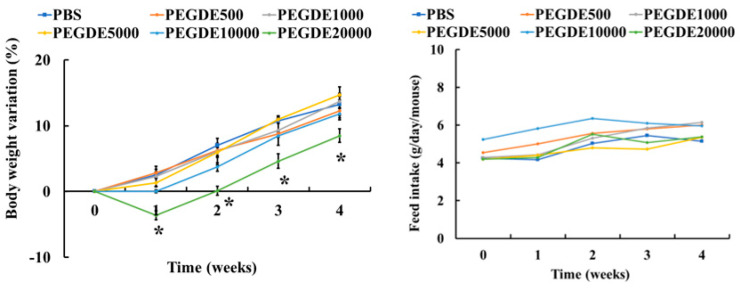
Changes in the body weight (%) and feed intake (g/day/mouse) of mice treated with Poly(ethylene glycol) diglycidyl ether (PEGDE). Body weights of mice were measured every 7 days after PEGDE injection. The feed intake in mice was measured every 7 days after PEGDE injection. Seven-week-old BALB/c male mice were grouped into PBS (control), PEGDE 500 μg/mouse, PEGDE 1000 μg/mouse, PEGDE 5000 μg/mouse, PEGDE 10,000 μg/mouse, and PEGDE 20,000 μg/mouse groups (*n* = 5 mice/group). * Indicates a significant difference vs. PBS control (* *p* < 0.05).

During the 28-day experimental period, no significant difference was found in the feed intake between the treatment groups (Figure 2). Mice injected with 0–10,000 μg of PEGDE showed no difference in body weight variation after 4 weeks (Figure 2). However, the body weight variation of mice injected with 20,000 μg of PEGDE was significantly lower than that of the other groups. The absolute and relative organ weights of all treatment groups (i.e., liver, kidney, and spleen weights) are shown in Table 3. No significant differences were observed in the absolute and relative organ weights between the groups. These data showed that subcutaneous PEGDE injection (up to 20,000 μg/mouse) in mice did not significantly exert toxicity in the three organs.

### 3.3. Histological Examination

**Figure 3:**
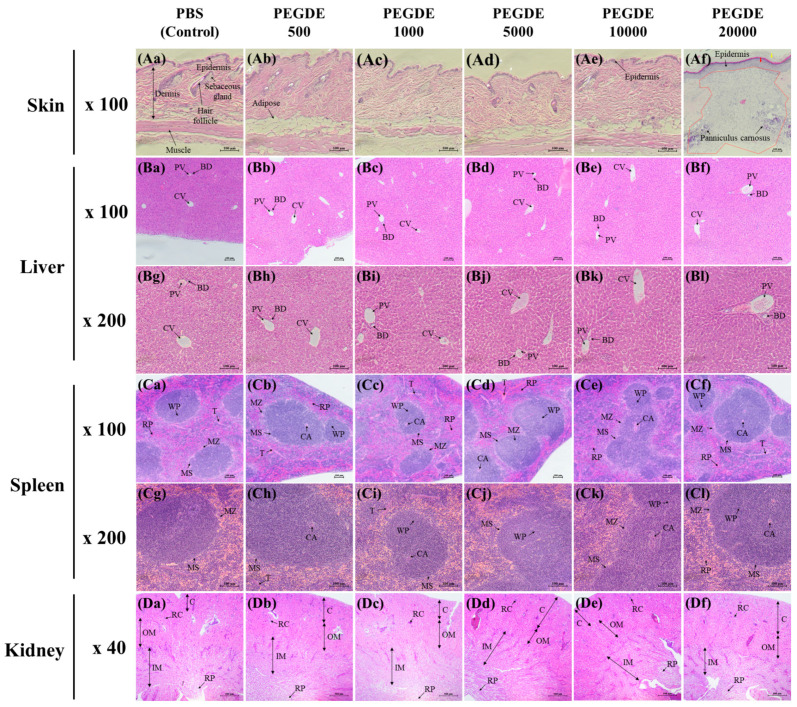
Histological analysis of mouse tissues. Skin, liver, spleen, and kidney sections were subjected to hematoxylin-eosin staining, followed by histopathological assessment by microscopy. Seven-week-old BALB/c male mice were grouped into PBS (control), PEGDE 500 μg/mouse, PEGDE 1000 μg/mouse, PEGDE 5000 μg/mouse, PEGDE 10,000 μg/mouse, and PEGDE 20,000 μg/mouse groups (*n* = 5 mice/group). (**Aa**–**Ae**) Histology images of skin sections at 100× magnification. (**Af**) Histology images of skin sections at 200× magnification. The red arrow indicates thickened epidermis, the yellow arrow indicates the detachment of keratin layer, and the orange-dotted line indicates closed wound. (**Ba**–**Bf**) Histology images of liver sections at 100× magnification. PV, portal vein; BD, bile duct; CV, central vein. (**Bg**–**Bl**) Histology images of liver sections at 200× magnification. (**Ca**–**Cf**) Histology images of spleen sections at 100x magnification. CA, central artery; MS, marginal sinus region; MZ, marginal zone; T, trabecula; RP, red pulp; WP, white pulp. (**Cg**–**Cl**) Histology images of spleen sections at 200× magnification. (**Da**–**Df**) Histology images of spleen sections at 40× magnification. C, cortex; OM, outer medulla; IM, inner medulla; RC, renal corpuscle; RP, renal papilla.

To investigate the toxicity of PEGDE in mouse tissues, histopathological examinations of the skin, liver, spleen, and kidneys were conducted. In the skin tissue, mice injected with up to 10,000 μg of PEGDE showed no histological difference in the thickness of epidermis, structure of panniculus carnosus, and dermis morphology, compared to PBS-injected mice (Figure 3(Aa)–(Ae)). No other lesions were observed in the same dermal samples (Figure 3(Aa)–(Ae)). However, mice injected with 20,000 μg of PEGDE exhibited a thickened epidermis (red arrow), interrupted panniculus carnosus, infiltration of inflammatory cells, detachment of the keratin layer (yellow arrow), and lack of hair follicles (re-epithelialized wounds) (Figure 3(Af)). Additionally, some skin tissues from this group of mice showed unhealed wounds containing granulation tissue and fibroblasts (Figure S1). Histological evaluations of liver tissue are shown in Figure 3B. The murine liver consists of a lobular structure, including central venules, portal veins, and small bile ducts. No evidence of abnormal appearance or histopathological alteration was observed in the hepatic tissue from all PEGDE-treated groups (Figure 3(Ba)–(Bl)). Histology images of spleen tissues from mice are shown in Figure 3C. In all mice groups, clearly defined red and white pulp regions divided by the marginal zone and marginal sinus region were observed (Figure 3(Ca–Cl)). These data indicate that subcutaneously injected PEGDE did not systemically affect the spleen. The renal histological structures showed normal histoarchitecture of renal glomeruli and regular renal tubules in all groups (Figure 3(Da–Df)). Moreover, disruption of the renal structure or infiltration of inflammatory cells in the medulla was not observed.

## 4. Discussion

PEGDE is widely used in polymer sciences for various purposes, mainly as a cross-linking agent for biomaterials (such as HA-based hydrogels and carboxymethyl cellulose-based hydrogels) [18,19]. Residual cross-linkers can be released from cross-linked HA chains during the production of hydrogels and when degraded inside the tissue [20]. The residual cross-linker molecules may be toxic unless they are bound to other molecules. For example, HA hydrogels cross-linked with 10 ppm of BDDE showed cytotoxicity in human foreskin fibroblasts [21]. However, toxicity assessment of PEGDE administered via the subcutaneous route has not been conducted in animal models. Therefore, the current study aimed to evaluate the toxicity of PEGDE in mice after a single subcutaneous injection.

Due to insufficient information on the toxicity of PEGDE administered subcutaneously, it is important to find an appropriate dose range in mice [22]. Accordingly, as a preliminary study, male mice were injected with 10,000 μg and 100,000 μg of PEGDE through the subcutaneous route at the paraspinal dorsum. Mice treated with 100,000 μg of PEGDE died 2–3 h after injection, whereas the mice treated with 10,000 μg of PEGDE survived until 28 days after the injection. Therefore, the highest dose of PEGDE for mouse survival was determined to be in the range of 10,000–100,000 μg. In the current study, the highest dose level was selected as 40,000 μg, and lower doses (0, 500, 1000, 5000, 10,000, and 20,000 μg) were selected to investigate dose-response relationships. Additionally, the duration of the experiment (28 days) was decided based on studies using HA hydrogel dermal fillers [23,24].

In the cage-side observation, we observed that PEGDE at concentrations of up to 5000 μg/mouse did not induce severe clinical responses in mice. However, mice injected with 40,000 μg of PEGDE showed decreased movement and abnormalities in gait, breathing, and appearance. The mice exhibited slight hindlimb paralysis, moving only with the forelimbs 3 h after injection. Therefore, this mouse group was euthanized. These results indicate that subcutaneous injection of PEGDE at 40,000 μg can cause acute clinical and systemic toxicity.

In the present study, mice subcutaneously injected with PEGDE (up to 20,000 μg/mouse) showed no mortality and difference in behavior pattern, appearance, gait, fur condition, and breathing abnormalities up to nine days after injection. However, at 10 days post-injection, mice in the high-dose groups (10,000 and 20,000 μg/mouse) had hair loss around the injection area, epidermal ulceration, and dull fur. Moreover, the size of skin ulcers of 10,000 μg PEGDE-injected mice increased until 17 days after injection. Subsequently, the disappearance of ulcers was confirmed by histopathological analysis of the skin tissue. Results of dermal histopathology evaluation showed no difference between the PEGDE group (10,000 μg) and the control (PBS) group. However, hair restoration at the injection site did not occur until the end of the study (28 days). In the 20,000 μg PEGDE-injected mice group, the epidermal ulcers did not disappear until the end of the study (28 days). Skin wounds are caused by various damaging agents and factors that result in disruption of skin continuity [25]. In previous studies, clinical observations of epidermal ulcers and hair loss around the injection site were similar to our results. For example, cedarwood oil applied dermally to F344/N rats and B6C3F1/N mice for 13 weeks induced ulceration, irritation, and thickened skin at a concentration of 12.5% [26]. Prolonged subcutaneous administration of agmatine, a polyamine produced by arginine decarboxylation, leads to ulceration and hair loss on the dorsal skin [27]. Our cage-side observation data indicate that PEGDE, subcutaneously injected at concentrations of more than 10,000 μg, can induce epidermal ulcers and hair loss around the injection site.

Changes in body weight and feed intake in animals usually occur due to the toxicity of the administered substances [28,29]. The feed intake of mice treated with PEGDE up to 20,000 μg showed no significant difference compared to that of the control (PBS) mice. However, in body weight variation, the body weight of mice injected with 20,000 μg of PEGDE group was lower than that of mice injected with lower PEGDE doses. These results demonstrated that subcutaneous injection of a high dose of PEGDE (20,000 μg) can decrease the rate of body weight gain without changing the rate of feed intake. Similar results were also found in other past studies. For example, mice developed nonalcoholic fatty acid liver disease exhibited reduction in body weight gain without changing food intake [30]. In addition, 6-week-old male mice orally administered with 400 mg/kg bw of carmoisine showed lower feed efficacy than control group [31]. These past studies and our data indicate that feed efficacy may be decreased in mice experiencing toxic responses. Additionally, the absolute and relative organ weights were measured to investigate the systemic effects of PEGDE on the major organs. Our data revealed that there was no significant difference in either absolute or relative organ weights, suggesting that there are no apparent systemic toxic responses in the liver, spleen, and kidneys.

Next, histopathological lesions were investigated to identify signs of systemic toxicity induced by PEGDE. Histological analysis revealed that skin sections of mice treated with PEGDE up to 10,000 μg/mouse showed no alteration in the thickness of epidermis, continuity of panniculus carnosus, presence of hair follicles, and attachment of the keratin layer. Mice injected with 10,000 μg of PEGDE exhibited hair loss and skin ulcers in cage-side observation. However, the mouse group showed no toxic histological responses. Histological analysis of skin lesions showed that the mice injected with 20,000 μg of PEGDE showed closed and re-epithelialized wounds, which are stages of the wound healing process. The difference in the severity of epidermal ulcers indicated that PEGDE induced dermal ulcers in a dose-dependent manner. The skin healing process includes the following four stages: homeostasis, inflammation, proliferation, and remodeling [32,33]. In the homeostasis stage, fibrin clot formation controls bleeding. The inflammatory phase activates the release of cytokines, stimulating the chemotaxis of neutrophils. In the proliferative phase, fibroblasts are formed around the wounds to synthesize granulation tissue, including procollagen, proteoglycans, and elastin. Then, the wound is closed and the remodeling stage begins, resulting in barrier recovery. In the current study, skin sections from the mice injected with 20,000 μg of PEGDE showed the appearance of closed wound in the remodeling stage, including the detachment of the keratin layer, thickened epidermis, disruption of the continuity of panniculus carnosus, and formation of a remodeling matrix. Additionally, the dermal section of a mouse injected with 20,000 μg of PEGDE exhibited characteristics of proliferation stage, such as the appearance of granulation tissue, fibroblasts, and newly formed epidermis (Figure S1). Normally, the proliferation phase of the wound healing process lasts 4–21 days [34]. Thus, this particular mouse may have a slower wound healing rate than that of other mice in the same group. In the liver, spleen, and kidney tissue sections, no abnormalities of histopathological lesions were observed in the PEGDE injection groups compared to the control group. Collectively, our histopathological data demonstrated that subcutaneously injected PEGDE provokes the formation of epidermal ulcers only at high injection doses (20,000 μg). Moreover, PEGDE up to 20,000 μg induces no histopathological alterations in the liver, spleen, and kidneys. The toxicity of PEGDE may be due to the two terminal epoxy groups in PEGDE which are highly reactive [35]. Epoxide rings of cross-linking agents such as PEGDE and BDDE react with the hydroxyl groups of HA that lead to the formation of ether bond connection [36]. However, unreacted epoxide rings can result in toxicity. For example, in cytotoxicity test using neutral red assay, six diepoxy compounds decreased cell viability in normal rabbit cornea epidermal cells and mouse fibroblasts (L929) [37]. Epoxides can bind with proteins and nucleic acids that lead to toxic effects including carcinogenesis and mutagenesis [38]. In addition, high concentration of PEGDE (>100 ppm) showed cytotoxicity in human keratinocyte cell line (HaCaT) and human dermal fibroblast cell line (HDF) by disrupting cell membrane, producing intracellular reactive oxygen species, and increasing mRNA expression of inflammatory cytokines [16]. Collectively, the toxicity of PEGDE in the current study was probably due to epoxy groups on PEGDE. To the best of our knowledge, our study is the first to report on the subcutaneous toxicity of PEGDE in mice. Our in vivo data suggest that a high dose of PEGDE injected (i.e., 10,000 and 20,000 μg) through the subcutaneous route can cause toxic responses in the dermal injection area. Furthermore, our data imply that PEGDE is safe up to a concentration of 5000 μg when injected subcutaneously.

## 5. Conclusions

Our data suggest that the lower doses of subcutaneously injected PEGDE (500, 1000, and 5000 μg per mouse) are relatively safe because mice did not show clinical abnormalities and histological lesions in the skin, liver, spleen, and kidneys. Higher doses of subcutaneously injected PEGDE (10,000, 20,000, and 40,000 μg per mouse) exerted toxicity in mice by causing epidermal ulcers, histological lesions, and clinical abnormalities depending on the dosage. This study will help determine the optimum concentration of PEGDE that can be administered via the subcutaneous route.

## Figures and Tables

**Table 1:** Experimental groups in the 28-day toxicity study of subcutaneous PEGDE injection.

Group	Injection Dose (μg PEGDE/mouse)	PEGDE Volume (μL)	PBS Volume (μL)	Total Volume (μL/mouse)
PBS	0	0	200	200
PEGDE500	500	0.439	199.561	200
PEGDE1000	1000	0.877	199.123	200
PEGDE5000	5000	4.386	195.614	200
PEGDE10000	10,000	8.772	191.228	200
PEGDE20000	20,000	17.544	182.456	200
PEGDE40000	40,000	35.088	164.912	200

Abbreviations: PEGDE, Poly(ethylene glycol) diglycidyl ether; PBS, phosphate-buffered saline.

**Table 2:** Cage-side observation after subcutaneous injection of PEGDE.

	Cage-Side Observation						
Sample	PBS	PEGDE					
PEGDE Dose (μg/mouse)	0	500	1000	5000	10,000	20,000	40,000
ppm	0	14	29	143	286	571	1143
Mortality	0 *	0	0	0	0	0	1
Morbidity	0	0	0	0	0	1	3
Appearance	0	0	0	0	1	1	3
Behavior pattern	0	0	0	0	0	0	2
Gait	0	0	0	0	0	0	3
Condition of the fur	0	0	0	0	1	1	2
Breathing abnormalities	0	0	0	0	0	0	2

* The grade of any observed signs was recorded. Signs were graded for severity, and the maximum grade was defined as 4. Grades were coded as none (grade 0), slight (grade 1), moderate (grade 2), severe (grade 3), or very severe (grade 4). For mortality, only the presence (grade 1) or absence (grade 0) of death was scored.

**Table 3:** Absolute and relative organ weights.

	Organ Weights					
Sample	PBS	PEGDE				
PEGDE Dose (μg/mouse)	0	500	1000	5000	10,000	20,000
ppm	0	14	29	143	286	571
Absolute organ weight						
Liver (g)	1.41 ± 0.03 ^a^	1.27 ± 0.05 ^a^	1.25 ± 0.01 ^a^	1.38 ± 0.06 ^a^	1.33 ± 0.07 ^a^	1.25 ± 0.05 ^a^
Kidney (g)	0.46 ± 0.07 ^a^	0.42 ± 0.01 ^a^	0.43 ± 0.02 ^a^	0.45 ± 0.01 ^a^	0.47 ± 0.01 ^a^	0.45 ±0.02 ^a^
Spleen (g)	0.13 ± 0.01 ^a^	0.13 ± 0.01 ^a^	0.13 ± 0.01 ^a^	0.14 ± 0.01 ^a^	0.13 ± 0.01 ^a^	0.13 ± 0.01 ^a^
Relative organ weight						
Liver (% of body weight)	5.67 ± 0.14 ^a^	5.21 ± 0.25 ^a^	5.01 ± 0.1 ^a^	5.46 ± 0.23 ^a^	5.26 ± 0.19 ^a^	5.14 ± 0.15 ^a^
Kidney (% of body weight)	1.86 ± 0.28 ^a^	1.74 ± 0.04 ^a^	1.72 ± 0.07 ^a^	1.79 ± 0.03 ^a^	1.86 ± 0.02 ^a^	1.84 ± 0.05 ^a^
Spleen (% of body weight)	0.51 ± 0.03 ^a^	0.52 ± 0.03 ^a^	0.51 ± 0.03 ^a^	0.6 ± 0.02 ^a^	0.53 ± 0.02 ^a^	0.53 ± 0.02 ^a^

Abbreviations: PEGDE, Poly(ethylene glycol) diglycidyl ether; PBS, phosphate-buffered saline. ^a^ Different letter indicate significant differences among PEGDE-treated groups (*p* < 0.05).

## Supplementary Materials

The following are available online at https://www.mdpi.com/article/10.3390/toxics9120354/s1, Figure S1: Histological analysis of mouse skin tissues.
