# Supplementary material for: Toxicity Assessment of a Single Dose of Poly(ethylene glycol) Diglycidyl Ether (PEGDE) Administered Subcutaneously in Mice
Source: Toxics. 2021 Dec 15;9(12):354. doi: 10.3390/toxics9120354 (PMC8708792; doi:10.3390/toxics9120354)
Supplement: Supplementary file 1 [file toxics-09-00354-s001.zip › Figure S1.pdf]

PEGDE
20000

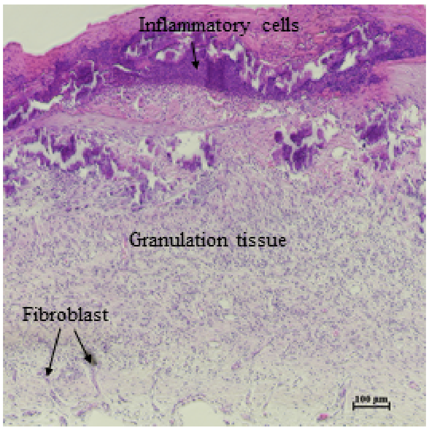

Figure S1. Histological analysis of mouse skin tissues. Seven-week-old BALB/c male mice were treated with 20,000 μg of PEGDE. Histology images of skin sections at 100x magnification.
